# Vaccination Papers Should Be Continued

**Published:** 1898-06

**Authors:** C. H. Oakes

**Affiliations:** Livermore Falls, Me.


### VACCINATION PAPERS SHOULD BE CONTINUED.

LIVERMORE FALLS, ME., April 29th, 1898.

EDITOR HOMŒOPATHIC PHYSICIAN:—In your March number you invite members of the profession to give full and free expression of views upon the further publication of evidence upon the subject of vaccination. For one I desire to express the hope that these articles will continue—that is, if we are getting a thoroughly reliable abstract of the testimony.

To many of us these facts are not otherwise easily accessible, and we cannot be too well prepared to face our opponents in their efforts at compulsory acceptance of this, "their joy and their pride," the sole relic of ostensible usefulness.

Sincerely yours,

C. H. OAKES.

